# Halogenated 1-Hydroxynaphthalene-2-Carboxanilides Affecting Photosynthetic Electron Transport in Photosystem II †

**DOI:** 10.3390/molecules22101709

**Published:** 2017-10-12

**Authors:** Tomas Gonec, Jiri Kos, Matus Pesko, Jana Dohanosova, Michal Oravec, Tibor Liptaj, Katarina Kralova, Josef Jampilek

**Affiliations:** 1 Department of Chemical Drugs, Faculty of Pharmacy, University of Veterinary and Pharmaceutical Sciences, Palackeho 1, Brno 61242, Czech Republic; jurd@email.cz; 2 Department of Pharmaceutical Chemistry, Faculty of Pharmacy, Comenius University, Odbojarov 10, Bratislava 83232, Slovakia; 3 Department of Environmental Ecology, Faculty of Natural Sciences, Comenius University, Ilkovicova 6, Bratislava 84215, Slovakia; matus.pesko@gmail.com; 4 Central Laboratories, Faculty of Chemical and Food Technology, Slovak University of Technology in Bratislava, Radlinskeho 9, Bratislava 81237, Slovakia; jana.dohanosova@stuba.sk (J.D.); tibor.liptaj@stuba.sk (T.L.); 5 Global Change Research Institute CAS, Belidla 986/4a, 60300 Brno, Czech Republic; oravec.m@czechglobe.cz; 6 Institute of Chemistry, Faculty of Natural Sciences, Comenius University, Ilkovicova 6, Bratislava 84215, Slovakia; kata.kralova@gmail.com

**Keywords:** hydroxynaphthalene-carboxamides, photosynthetic electron transport (PET) inhibition, spinach chloroplasts, structure-activity relationships

## Abstract

Series of seventeen new multihalogenated 1-hydroxynaphthalene-2-carboxanilides was prepared and characterized. All the compounds were tested for their activity related to the inhibition of photosynthetic electron transport (PET) in spinach (*Spinacia oleracea* L.) chloroplasts. 1-Hydroxy-*N*-phenylnaphthalene-2-carboxamides substituted in the anilide part by 3,5-dichloro-, 4-bromo-3-chloro-, 2,5-dibromo- and 3,4,5-trichloro atoms were the most potent PET inhibitors (IC_50_ = 5.2, 6.7, 7.6 and 8.0 µM, respectively). The inhibitory activity of these compounds depends on the position and the type of halogen substituents, i.e., on lipophilicity and electronic properties of individual substituents of the anilide part of the molecule. Interactions of the studied compounds with chlorophyll *a* and aromatic amino acids present in pigment-protein complexes mainly in PS II were documented by fluorescence spectroscopy. The section between P_680_ and plastoquinone Q_B_ in the PET chain occurring on the acceptor side of PS II can be suggested as the site of action of the compounds. The structure-activity relationships are discussed.

## 1. Introduction

In spite of the fact that various classes of herbicides are known and currently can be classified according to about 20 different modes of action [1], over 50% of marketed herbicides act by reversible binding to photosystem II (PS II) [2]. This effect results in the interruption of the photosynthetic electron transport (PET) [3,4,5]. PS II uses light energy to drive two chemical reactions: the oxidation of water and the reduction of plastoquinone. The following redox components of PS II participate in the transfer of electrons from H_2_O to the plastoquinone pool: water oxidizing manganese cluster (Mn)_4_ and redox-active tyrosine (Tyr_z_) situated on the donor side of PS II, reaction centre chlorophyll (P680) and components situated on the acceptor side of PS II, namely pheophytin and two plastoquinone molecules, Q_A_ and Q_B_. While the plastoquinone molecule Q_A_ acting as a one-electron acceptor is permanently bound to PS II, the plastoquinone molecule Q_B_ acting as a two-electron acceptor is loosely bound at the Q_B_-site, and reduced plastoquinone unbinds from the reaction centre and diffuses in the hydrophobic core of the membrane, whereby Q_B_-binding site will be occupied by an oxidized plastoquinone molecule [6]. Herbicides belonging to photosystem (PS) II inhibitors inhibit photosynthetic electron transport (PET) by binding to the Q_B_-binding niche on the D_1_ protein of the PS II complex in chloroplast thylakoid membranes, which results in the inhibition of photosynthetic electron transport (PET) from Q_A_ to Q_B_, blockage of CO_2_ fixation and inhibition of ATP production. Many QSAR studies of PS II inhibitors with diverse chemical structures have emphasized the hydrophobic nature of the binding domain, with lipophilicity being the dominant determinant of Hill inhibition activity [7], e.g., the review paper of Lambreva et al. [8] presents a compendious overview of the recent improvements in the understanding of the structure and function of PS II donor side with a focus on the interactions of the plastoquinone cofactors with the surrounding environment and operational features. PET inhibitors in the functional dissection of the photosynthetic electron transport system were summarized in the review paper of Trebst [9], and a comprehensive overview of synthetic photosynthetic inhibitors and those based on natural products was published by Teixeira et al. [10].

Many herbicides acting as PET inhibitors in PS II containing an amide and/or a carbamate moiety, which is able to form hydrogen bonds between the CONH/NHCOO group of amides/carbamates and the target proteins in photosynthetic centres of thylakoid membranes resulting in changes in protein conformation and finally in PET inhibition, have been shown to be the most potent type of this class of herbicides [11,12,13]. Both moieties can be substituted, and thus, their properties can be modified [14,15]. Amides are considered to be PS II inhibitors causing displacement of Q_B_ from its binding pocket in the D_1_ protein [16], and halogen substituents were found to contribute to enhanced PET-inhibiting activity [10,11,12,17,18,19,20,21,22,23]. Our team has investigated effects of various amides [11,16,18,19,20,21,22,24,25,26,27,28] or carbamates [29,30,31,32,33,34] on biological systems, in particular, as anti-invasive agents for, a long time. In addition, some of previously published agents showed herbicidal properties associated with adverse effects on PS II [12,13,16,18,19,20,21,22,23,24,28,29,33].

In the context of the above-mentioned facts, new di- and trichlorinated and brominated 1-hydroxynaphthalene-2-carboxanilides were prepared and characterized and tested for their activity related to the PET inhibition in spinach (*Spinacia oleracea* L.) chloroplasts. The results were completed by seven recently published unsubstituted and monochlorinated and brominated derivatives [11], and the structure–activity relationships (SAR) of all the mentioned compounds are discussed.

## 2. Results and Discussion

### 2.1. Chemistry

**Scheme 1:**
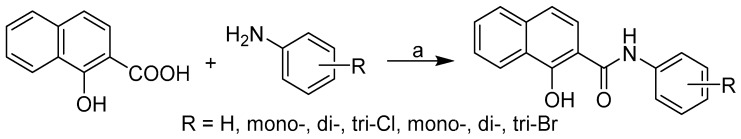
Synthesis of ring-substituted 1-hydroxynaphthalene-2-carboxanilides **1**–**24**. *Reagents and conditions*: (a) PCl_3_, chlorobenzene, microwave synthesis.

The discussed anilides were synthesized using microwave-assisted synthesis as described in Gonec et al. [11]. All the studied compounds were prepared according to Scheme 1. The condensation of 1-hydroxy-2-naphthoic acid and ring-substituted aniline using phosphorus trichloride in chlorobenzene gave a series of twenty-four 1-hydroxynaphthalene-2-carboxanilides **1**–**24**.

Physicochemical descriptors—lipophilicity of the compounds expressed as log *P* and electronic σ parameters of individual anilides—were calculated for all the investigated compounds by means of the ACD/Percepta ver. 2012 program, see Table 1. Log *P* values ranged from 4.52 (unsubstituted anilide **1**) to 6.34 (2,4,6-Br substituted compound **20**). In general, it can be stated that the lipophilicity is rather high; nevertheless, the recommended log *P* value for agrochemicals is ≤5 [35]. Beside unsubstituted compound **1,** monohalogenated anilides **2**–**7** expressed the lowest lipophilicity, while trisubstituted derivatives **14**–**16** and **20** possessed the highest log *P* values, and in general brominated compounds showed higher lipophilicity than chlorinated. For individual *N*-aryl parts of the anilides also electronic properties expressed as electronic σ constants of the whole substituted phenyl ring were predicted; they ranged from 0.60 (compound **1**) to 1.56 (2,4,5-Cl substituted derivative **16**).

### 2.2. Inhibition of Photosynthetic Electron Transport (PET) in Spinach Chloroplasts

In this contribution, variously chlorinated and brominated anilides and their PET-inhibiting activity are discussed. The PET-inhibiting activity was expressed by IC_50_ value (compound concentration causing 50% inhibition of PET), see Table 1. The activity of the studied compounds was compared with diuron (3-(3,4-dichlorophenyl)-1,1-dimethylurea; DCMU), a commercial PET inhibitor. The obtained activity showed a wide range of PET inhibition, from 5.2 µM (compound **13**, R = 3,5-Cl) to 531 µM (compound **9**, R = 2,4-Cl). In general, it can be stated that chlorinated derivatives demonstrated higher potency than bromine derivatives. 2,4-Disubstituted compounds, i.e., **9** (R = 2,4-Cl), **17** (R = 2,4-Br), **21** (R = 2-Br-4-Cl), **22** (R = 2-Cl-4-Br), and one 2,3-disubstituted compound **8** (R = 2,3-Cl) showed limited aqueous solubility compared with 2,5-, 2,6-, 3,4- and 3,5-disubstituted derivatives or the rest of compounds. Therefore, these compounds are excluded from the SAR discussion and this is illustrated on the figures by empty symbols, see below. It should be noted that the highest potency with IC_50_ = 5.2 µM was shown by symmetrically disubstituted compound **13** (R = 3,5-Cl), while the trisubstituted analogue—compound **16** (R = 3,4,5-Cl)—expressed already PET-inhibiting activity IC_50_ = 8.0 µM, and mono-chlorinated derivatives **3** and **4** showed IC_50_ of 7.9 µM (R = 3-Cl) and 10.8 µM (R = 4-Cl), respectively. Thus, it seems that physicochemical properties resulting from C′_(3,5)_ position of both chlorine atoms are preferable.

**Figure 1:**
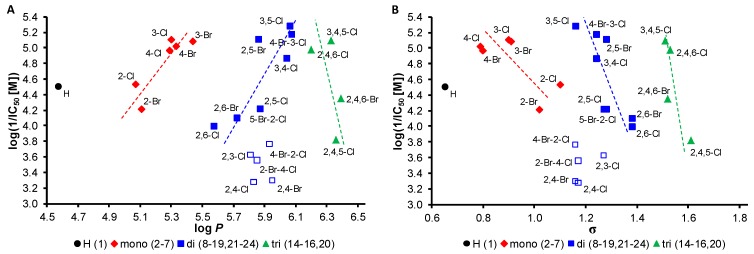
Dependence of PET-inhibiting activity log(1/IC_50_ [M]) of all discussed compounds **1**–**24** in spinach chloroplasts on lipophilicity expressed as log *P* (**A**) and electronic σ parameters of whole *N*-aryl part of individual anilides (**B**). Empty squares are not involved in SAR discussion.

The dependences of the PET-inhibiting activity on the lipophilicity of the discussed mono-, di- and tri-*N*-aryl substituted anilides are shown in Figure 1A. Similar trends for mono- and disubstituted compounds can be observed—the PET-inhibiting activity increases with increasing lipophilicity, while an opposite trend can be found for trisubstituted derivatives. Thus, it seems that for PET-inhibiting activity, a lipophilicity optimum is in the range of log *P* from 6.01 (compound **13**) to 6.28 (compound **16**), see Figure 1A. It is important to note that a PET inhibition depends also on the electronic effects of individual substituents within series of different PET inhibitors [11,12,21,22,23]. The dependences of the PET-inhibiting activity on the electron σ parameters of the whole *N*-aryl part of individual anilides are illustrated in Figure 1B. Practically the same trends can be found for mono-, di- and trisubstituted derivatives; the PET inhibition sharply decreases with the increasing electron-withdrawing effect of individual anilides (i.e., with high amide bond electron-deficiency) as follows: compound **3** (R = 3-Cl, σ = 0.85, IC_50_ = 7.9 µM) >>> compound **5** (R = 2-Br, σ = 0.97, IC_50_ = 61.9 µM); compound **13** (R = 3,5-Cl, σ = 1.11, IC_50_ = 5.2 µM) >>> compound **11** (R = 2,6-Cl, σ = 1.33, IC_50_ = 100.5 µM); compound **16** (R = 3,4,5-Cl, σ = 1.46, IC_50_ = 8.0 µM) >>> compound **14** (R = 2,4,5-Cl, σ = 1.56, IC_50_ = 153.2 µM).

1,5-Diphenylcarbazide (DPC), an efficient artificial electron donor to the inactive oxygen-evolving complex (OEC) of PS II acting in Z/D intermediate situated on the donor side of PS II can be used for a more precise determination of the site of action of the tested compounds in the PET chain [36,37,38]. This intermediate ensures electron transport from the OEC to the core of PS II (P680) [39]. Consequently, an addition of DPC can completely restore the activity of chloroplasts, which was inhibited by PET inhibitors acting on the donor side of PS II. However, the addition of DPC to chloroplasts, the activity of which was inhibited by tested compounds approximately up to 80% (i.e., corresponding to 20% activity of the control), caused gradual PET restoration with increasing DPC concentration, and complete activity restoration was obtained only with 12-fold higher DPC concentration compared to the concentration of the applied PET inhibitor. Based on this finding, it could be suggested that the site of inhibitory action of the halogenated 1-hydroxynaphthalene-2-carboxanilides is situated on the acceptor side of PS II, at the section between P680 (primary donor of PS II) and Q_B_. The complete restoration of PET using DPC concentration exceeding that of the applied inhibitor by more than one order of magnitude indicates that, due to direct interaction of DPC with the herbicide binding niche, these PET inhibitors were displaced from their binding site, as was similarly shown for atrazine [40] or metribuzin [41]. The plastoquinone Q_B_ at the acceptor side of PS II was found to be the site of inhibitory action of *N*-benzylpyrazine-2-carboxamides [42] and *N*-alkoxyphenyl-3-hydroxy-naphthalene-2-carboxamides [16] as well as ring-substituted salicylanilides, carbamoylphenyl-carbamates [29] and 8-hydroxyquinoline-2-carboxamides [23].

**Figure 2:**
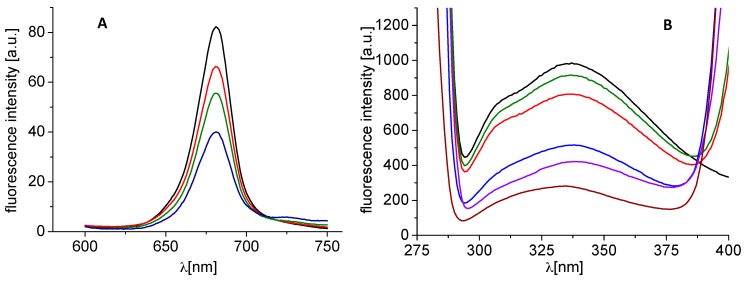
Fluorescence emission spectra of chlorophyll *a* in untreated spinach chloroplasts in presence of compound **23** (R = 5-Br-2-Cl; c = 0, 56, 220, and 431 μM; curves from top to bottom); excitation wave length λ_ex_ = 436 nm, chlorophyll concentration 10 mg/L (**A**) and fluorescence emission spectra of aromatic amino acids in suspension of spinach chloroplasts without and with compound **23** (c = 0, 5.9, 11.8, 23.6, 29.5 and 47.2 μM; curves from top to bottom); excitation wave length λ = 275 nm, chlorophyll concentration 10 mg/L (**B**).

In isolated chloroplasts, the interaction of a PET inhibitor with chlorophyll *a* (Chl*a*) molecules situated in pigment–protein complexes in PS II is reflected in changes of the intensity of Chl*a* fluorescence emission band at 686 nm. A gradual decrease of the intensity of this Chl*a* emission band at 686 nm with the increasing concentration of *N*-(5-bromo-2-chlorophenyl)-1-hydroxynaphthalene-2-carboxamide (**23**) (Figure 2A) indicates increased perturbation of Chl*a*–protein complexes in the thylakoid membrane [43] by this PET inhibitor. A similar Chl*a* fluorescence decrease in spinach chloroplasts was observed previously for several PET inhibitors, such as substituted benzanilides [44], *N*-benzylpyrazine-2-carboxamides [42], 3,5-dibromo-2-hydroxy-*N*-phenylbenzamides [45], ring-substituted 8-hydroxyquinoline-2-carboxamides [23], 3-hydroxynaphthalene-2-carboxamides [21] and 2-alkylpyridine-4-carboxylic acids [46].

In addition, the interaction of PET inhibitors with aromatic amino acids (AAA), mainly tryptophan and tyrosine, occurring in photosynthetic proteins situated mainly in PS II can be investigated using the fluorescence method. In the presence of the tested halogenated 1-hydroxynaphthalene-2-carboxanilides, quenching of AAA fluorescence at 334 nm was observed, which showed an increase with increasing concentration of PET inhibitors, which is presented in Figure 2B for the model compound **23**. It could be assumed that the decrease in fluorescence is caused by a change in the environment of AAAs upon interaction with the tested compounds. The quenching of the AAA fluorescence in the presence of various PET inhibitors such as ring-substituted 8-hydroxyquinoline-2-carboxamides [23], 5-bromo- and 3,5-dibromo-2-hydroxy-*N*-phenylbenzamides [45], *N*-substituted 5-amino-6-methylpyrazine-2,3-dicarbonitriles [47] and 2-substituted 6-fluoro-benzothiazoles [48] was observed previously.

## 3. Materials and Methods

### 3.1. General Information

All reagents were purchased from Merck (Sigma-Aldrich, St. Louis, MO, USA) and Alfa (Alfa-Aesar, Ward Hill, MA, USA). Reactions were performed using a CEM Discover SP microwave reactor (CEM, Matthews, NC, USA). The melting points were determined on a Kofler hot-plate apparatus HMK (Franz Kustner Nacht KG, Dresden, Germany) and are uncorrected. Infrared (IR) spectra were recorded on a Smart MIRacle™ ATR ZnSe for Nicolet™ Impact 410 Fourier-transform IR spectrometer (Thermo Scientific, West Palm Beach, FL, USA). The spectra were obtained by the accumulation of 256 scans with 2 cm^−1^ resolution in the region of 4000–650 cm^−1^. All ^1^H- and ^13^C-NMR spectra were recorded on an Agilent VNMRS 600 MHz system (600 MHz for ^1^H and 150 MHz for ^13^C, Agilent Technologies, Santa Clara, CA, USA) in dimethyl sulfoxide-*d*_6_ (DMSO-*d*_6_). ^1^H and ^13^C chemical shifts (δ) are reported in ppm. The prepared compounds were analysed by an ultra-high performance liquid chromatograph (Dionex UltiMate^®^ 3000 Liquid Chromatography Systems, Thermo Scientific) coupled with a diode-array detector (DAD, Thermo Scientific) and a high resolution Hybrid Ion Trap-Orbitrap XL™ Fourier transform mass spectrometer (HRMS, Thermo Scientific). A chromatographic column Hypersil Gold (Thermo Scientific), C18 3 μm, 2.1 mm × 50 mm, was used. The mixture of MeCN-HPLC grade (20.0%) and H_2_O-HPLC grade with 0.1% AcOH (80.0%) was used as a mobile phase. The total flow of the column was 0.3 mL/min; the column temperature was 30 °C; and the time of analysis was 10 min. The records were evaluated from the DAD and HRMS-Orbitrap. The wavelengths of 210, 254, 272, 274, 331 nm and 190–800 nm were monitored. The peaks in the chromatogram of the solvent (blank) were deducted from the peaks in the chromatogram of the sample solution. The purity of individual compounds was determined from the area peaks in the chromatogram of the sample solution at wavelength of 210 nm. MS and MS^n^ were performed using the HRMS-Orbitrap equipped with a HESI II (heated electrospray ionization) source.

### 3.2. Synthesis

#### General Procedure for Synthesis of *N*-(substituted phenyl)-1-hydroxynaphthalene-2-carboxamides **1**–**24**

1-Hydroxynaphthalene-2-carboxylic acid (5.3 mmol) and the corresponding substituted aniline (5.3 mmol) were suspended in 30 mL of dry chlorobenzene. Phosphorous trichloride (2.65 mmol) was added dropwise, and the reacting mixture was heated in the microwave reactor at maximal allowed power 500 W and 130 °C, using infrared flask-surface control of temperature, for 15 min. The solvent was evaporated under reduced pressure, the solid residue washed with 2 M HCl, and the crude product was recrystallized from aqueous ethanol. All the studied compounds are presented in Table 1.

Described anilides **1**–**7** were characterized recently by Gonec et al. [11].

*N-(2,3-Dichlorophenyl)-1-hydroxynaphthalene-2-carboxamide* (**8**). Yield 59%; m.p. 199–201 °C; HPLC purity 98.32%; IR (cm^−1^): 3418, 1634, 1580, 1532, 1504, 1450, 1408, 1390, 1358, 1327, 1302, 1271, 1243, 1205, 1190, 1150, 1089, 1044, 878, 824, 799, 779, 770, 755, 738, 722, 700; ^1^H-NMR (DMSO-*d*_6_), δ: 13.67 (br. s, 1H), 10.84 (s, 1H), 8.32 (d, 1H, *J* = 7.8 Hz), 8.08 (d, 1H, *J* = 8.7 Hz), 7.93 (d, 1H, *J* = 8.2 Hz), 7.66–7.72 (m, 2H), 7.63 (dd, 1H, *J* = 8.2, *J* = 1.4 Hz), 7.60 (ddd, 1H, *J* = 8.2, *J* = 6.9, *J* = 1.4 Hz), 7.50 (d, 1H, *J* = 1.2 Hz), 7.47 (t, 1H, *J* = 7.8 Hz); ^13^C-NMR (DMSO-*d*_6_), δ: 169.22, 159.37, 136.23, 136.14, 132.06, 129.24, 128.49, 128.44, 128.26, 127.61, 127.27, 126.06, 124.66, 123.23, 123.12, 118.41, 107.61; HR-MS: [M − H]^+^ calculated 330.00831 *m*/*z*, found 330.01007 *m*/*z*.

*N-(2,4-Dichlorophenyl)-1-hydroxynaphthalene-2-carboxamide* (**9**). Yield 76%; m.p. 181–184 °C; HPLC purity 98.45%; IR (cm^−1^): 3422, 1629, 1580, 1524, 1503, 1472, 1456, 1391, 1331, 1300, 1243, 1211, 1098, 867, 818, 796, 787, 761, 715; ^1^H-NMR (DMSO-*d*_6_), δ: 13.67 (br. s, 1H), 10.74 (s, 1H), 8.32 (d, 1H, *J* = 8.2 Hz), 8.08 (d, 1H, *J* = 8.7 Hz), 7.93 (d, 1H, *J* = 8.2 Hz), 7.79 (d, 1H, *J* = 2.3 Hz), 7.75 (d, 1H, *J* = 8.7 Hz), 7.68 (ddd, 1H, *J* = 8.1, *J* = 7.0, *J* = 0.9 Hz), 7.59 (ddd, 1H, *J* = 8.2, *J* = 6.9, *J* = 1.4 Hz), 7.53 (dd, 1H, *J* = 8.7, *J* = 2.3 Hz), 7.49 (d, 1H, *J* = 8.7 Hz); ^13^C-NMR (DMSO-*d*_6_), δ: 169.17, 159.31, 136.13, 133.40, 131.40, 130.70, 129.70, 129.22, 129.16, 127.87, 127.60, 126.04, 124.66, 123.24, 123.11, 118.40, 107.67; HR-MS: [M − H]^+^ calculated 330.00831 *m*/*z*, found 330.01007 *m*/*z*.

*N-(2,5-Dichlorophenyl)-1-hydroxynaphthalene-2-carboxamide* (**10**). Yield 69%; m.p. 194–196 °C; HPLC purity 99.08%; IR (cm^−1^): 3418, 1632, 1583, 1525, 1502, 1471, 1407, 1392, 1320, 1280, 1239, 1209, 1151, 1089, 1047, 916, 876, 806, 786, 757, 725; ^1^H-NMR (DMSO-*d*_6_), δ: 13.47 (s, 1H), 10.83 (s, 1H), 8.33 (d, 1H, *J* = 8.6 Hz), 8.05–8.10 (m, 1H), 7.95 (d, 1H, *J* = 2.6 Hz), 7.93 (d, 1H, *J* = 8.2 Hz), 7.68 (ddd, 1H, *J* = 8.2, *J* = 6.9, *J* = 1.3 Hz), 7.65 (d, 1H, *J* = 8.9 Hz), 7.60 (ddd, 1H, *J* = 8.3, *J* = 7.0, *J* = 1.2 Hz), 7.51 (d, 1H, *J* = 8.9 Hz), 7.42 (dd, 1H, *J* = 8.7, *J* = 2.5 Hz); ^13^C-NMR (DMSO-*d*_6_), δ: 168.70, 158.81, 136.15, 135.64, 131.69, 130.92, 129.17, 127.67, 127.61, 127.40, 127.38, 126.03, 124.71, 123.48, 123.13, 118.58, 108.25; HR-MS: [M − H]^+^ calculated 330.00831 *m*/*z*, found 330.00986 *m*/*z*.

*N-(2,6-Dichlorophenyl)-1-hydroxynaphthalene-2-carboxamide* (**11**). Yield 59%; m.p. 199–202 °C; HPLC purity 98.82%; IR (cm^−1^): 3309, 1635, 1616, 1596, 1568, 1529, 1499, 1448, 1435, 1383, 1331, 1283, 1252, 1209, 944, 796, 766, 721, 701; ^1^H-NMR (DMSO-*d*_6_), δ: 13.85 (s, 1H), 10.76 (s, 1H), 8.28–8.33 (m, 1H), 8.11 (d, 1H, *J* = 8.9 Hz), 7.93 (d, 1H, *J* = 8.2 Hz), 7.69 (ddd, 1H, *J* = 8.1, *J* = 6.9, *J* = 1.2 Hz), 7.65 (d, 2H, *J* = 8.2 Hz), 7.60 (ddd, 1H, *J* = 8.1, *J* = 7.0, *J* = 1.0 Hz), 7.50 (d, 1H, *J* = 8.6 Hz), 7.47 (t, 1H, *J* = 8.2 Hz); ^13^C-NMR (DMSO-*d*_6_), δ: 169.90, 160.22, 136.15, 134.14, 132.10, 130.02, 129.37, 128.69, 127.58, 126.12, 124.56, 123.07, 122.72, 118.23, 106.37; HR-MS: [M − H]^+^ calculated 330.00831 *m*/*z*, found 330.00943 *m*/*z*.

*N-(3,4-Dichlorophenyl)-1-hydroxynaphthalene-2-carboxamide* (**12**). Yield 84%; m.p. 195–197 °C; HPLC purity 99.14%; IR (cm^−1^): 3429, 1628, 1601, 1584, 1574, 1527, 1504, 1467, 1390, 1320, 1298, 1274, 1247, 1210, 1144, 1127, 1088, 1026, 939, 871, 797, 785, 759, 729; ^1^H-NMR (DMSO-*d*_6_), δ: 13.85 (s, 1H), 10.76 (s, 1H), 8.28–8.33 (m, 1H), 8.11 (d, 1H, *J* = 8.9 Hz), 7.93 (d, 1H, *J* = 8.2 Hz), 7.69 (ddd, 1H, *J* = 8.1, *J* = 6.9, *J* = 1.2 Hz), 7.65 (d, 2H, *J* = 8.2 Hz), 7.60 (ddd, 1H, *J* = 8.1, *J* = 7.0, *J* = 1.0 Hz), 7.50 (d, 1H, *J* = 8.6 Hz), 7.47 (t, 1H, *J* = 8.2 Hz); ^13^C-NMR (DMSO-*d*_6_), δ: 169.56, 159.90, 137.90, 136.06, 130.86, 130.52, 129.28, 127.47, 126.21, 126.00, 124.56, 123.09, 123.00, 122.94, 121.68, 117.99, 107.42; HR-MS: [M − H]^+^ calculated 330.00831 *m*/*z*, found 330.00970 *m*/*z*.

*N-(3,5-Dichlorophenyl)-1-hydroxynaphthalene-2-carboxamide* (**13**). Yield 80%; m.p. 190–194 °C; HPLC purity 99.09%; IR (cm^−1^): 3431, 1626, 1585, 1568, 1526, 1504, 1443, 1407, 1387, 1300, 1267, 1204, 1144, 1086, 882, 823, 787, 755, 723; ^1^H-NMR (DMSO-*d*_6_), δ: 13.48 (br. s, 1H), 10.61 (s, 1H), 8.31 (d, 1H, *J* = 8.2 Hz), 8.04 (d, 1H, *J* = 8.9 Hz), 7.91 (d, 1H, *J* = 8.2 Hz), 7.88 (d, 2H, *J* = 1.6 Hz), 7.68 (ddd, 1H, *J* = 8.1, *J* = 7.0, *J* = 1.3 Hz), 7.58 (ddd, 1H, *J* = 8.1, *J* = 7.0, *J* = 1.0 Hz), 7.48 (d, 1H, *J* = 8.9 Hz), 7.39 (t, 1H, *J* = 1.8 Hz); ^13^C-NMR (DMSO-*d*_6_), δ: 169.69, 159.91, 140.23, 136.12, 133.92, 129.37, 127.50, 126.06, 124.56, 123.70, 123.13, 122.94, 119.74, 118.08, 107.45; HR-MS: [M − H]^+^ calculated 330.00831 *m*/*z*, found 330.00958 *m*/*z*.

*1-Hydroxy-N-(2,4,5-trichlorophenyl)naphthalene-2-carboxamide* (**14**). Yield 77%; m.p. 211–215 °C; HPLC purity 98.56%; IR (cm^−1^): 3422, 1630, 1574, 1514, 1501, 1470, 1446, 1392, 1367, 1320, 1287, 1269, 1242, 1205, 1146, 1088, 1068, 947, 884, 829, 876, 797, 785, 760, 715, 678; ^1^H-NMR (DMSO-*d*_6_), δ: 13.39 (br. s, 1H), 10.90 (s, 1H), 8.33 (d, 1H, *J* = 8.7 Hz), 8.18 (s, 1H), 8.06 (d, 1H, *J* = 8.7 Hz), 8.03 (s, 1H), 7.93 (d, 1H, *J* = 7.8 Hz), 7.68 (ddd, 1H, *J* = 8.0, *J* = 6.9, *J* = 1.4 Hz), 7.60 (ddd, 1H, *J* = 8.2, *J* = 6.9, *J* = 1.4 Hz), 7.51 (d, 1H, *J* = 8.7 Hz); ^13^C-NMR (DMSO-*d*_6_), δ: 168.63, 158.72, 136.19, 134.59, 130.67, 130.05, 129.24, 129.10, 128.55, 128.33, 127.64, 126.09, 124.71, 123.52, 123.16, 118.68, 108.30; HR-MS: [M − H]^+^ calculated 363.96934 *m*/*z*, found 363.97119 *m*/*z*.

*1-Hydroxy-N-(2,4,6-trichlorophenyl)naphthalene-2-carboxamide* (**15**). Yield 75%; m.p. 214–216 °C; HPLC purity 98.91%; IR (cm^−1^): 3303, 1631, 1616, 1597, 1556, 1524, 1500, 1468, 1445, 1407, 1385, 1320, 1278, 1241, 1211, 1189, 1154, 1142, 942, 851, 832, 796, 763, 713; ^1^H-NMR (DMSO-*d*_6_), δ: 13.73 (br. s, 1H), 10.79 (s, 1H), 8.30 (d, 1H, *J* = 8.7 Hz), 8.09 (d, 1H, *J* = 9.2 Hz), 7.93 (d, 1H, *J* = 8.2 Hz), 7.89 (s, 2H), 7.70 (ddd, 1H, *J* = 8.2, *J* = 6.9, *J* = 1.4 Hz), 7.60 (ddd, 1H, *J* = 8.2, *J* = 7.1, *J* = 1.1 Hz), 7.50 (d, 1H, *J* = 9.2 Hz); ^13^C-NMR (DMSO-*d*_6_), δ: 169.92, 160.22, 136.19, 134.96, 133.31, 131.54, 129.46, 128.50, 127.61, 126.18, 124.53, 123.09, 122.68, 118.33, 106.27; HR-MS: [M − H]^+^ calculated 363.96934 *m*/*z*, found 363.97119 *m*/*z*.

*1-Hydroxy-N-(3,4,5-trichlorophenyl)naphthalene-2-carboxamide* (**16**). Yield 71%; m.p. 245–248 °C; HPLC purity 99.28%; IR (cm^−1^): 3430, 1627, 1598, 1575, 1515, 1501, 1467, 1432, 1380, 1356, 1309, 1287, 1267, 1244, 1205, 1150, 1076, 959, 882, 870, 822, 797, 787, 757, 723, 682; ^1^H-NMR (DMSO-*d*_6_), δ: 13.41 (br. s, 1H), 10.64 (s, 1H), 8.29 (d, 1H, *J* = 8.2 Hz), 8.08 (s, 2H), 8.02 (d, 1H, *J* = 9.2 Hz), 7.90 (d, 1H, *J* = 8.2 Hz), 7.65–7.70 (m, 1H), 7.55–7.60 (m, 1H), 7.47 (d, 1H, *J* = 8.7 Hz); ^13^C-NMR (DMSO-*d*_6_), δ: 169.68, 159.95, 173.99, 136.14, 132.69, 129.42, 127.51, 126.08, 124.70, 124.55, 123.14, 122.86, 121.44, 118.12, 107.38; HR-MS: [M − H]^+^ calculated 363.96934 *m*/*z*, found 363.97119 *m*/*z*.

*N-(2,4-Dibromophenyl)-1-hydroxynaphthalene-2-carboxamide* (**17**). Yield 69%; m.p. 185–188 °C; HPLC purity 99.71%; IR (cm^−1^): 3410, 1630, 1575, 1521, 1502, 1471, 1456, 1410, 1389, 1378, 1358, 1328, 1300, 1236, 1209, 1151, 1085, 943, 866, 819, 795, 786, 760, 718, 694; ^1^H-NMR (DMSO-*d*_6_), δ: 13.72 (br. s, 1H), 10.69 (s, 1H), 8.31 (d, 1H, *J* = 8.2 Hz), 8.07 (d, 1H, *J* = 8.7 Hz), 8.03 (d, 1H, *J* = 2.3 Hz), 7.93 (d, 1H, *J* = 7.8 Hz), 7.62–7.71 (m, 3H) 7.57–7.62 (m, 1H), 7.49 (d, 1H, *J* = 9.2 Hz); ^13^C-NMR (DMSO-*d*_6_), δ: 169.22, 159.45, 136.12, 135.28, 134.72, 131.31, 130.43, 129.23, 127.60, 126.05, 124.65, 123.16, 123.11, 121.83, 119.82, 118.34, 107.55; HR-MS: [M − H]^+^ calculated 417.90727 *m*/*z*, found 417.90927 *m*/*z*.

*N-(2,5-Dibromophenyl)-1-hydroxynaphthalene-2-carboxamide* (**18**). Yield 72%; m.p. 198–200 °C; HPLC purity 98.82%; IR (cm^−1^): 3393, 1642, 1630, 1576, 1522, 1502, 1467, 1401, 1390, 1357, 1323, 1284, 1237, 1206, 1150, 1086, 1027, 943, 898, 871, 788, 750, 725; ^1^H-NMR (DMSO-*d*_6_), δ: 13.58 (br. s, 1H), 10.78 (s, 1H), 8.33 (d, 1H, *J* = 8.2 Hz), 8.07 (d, 1H, *J* = 9.2 Hz), 7.99 (d, 1H, *J* = 2.3 Hz), 7.93 (d, 1H, *J* = 8.2 Hz), 7.73 (d, 1H, *J* = 8.7 Hz), 7.66–7.71 (m, 1H) 7.57–7.62 (m, 1H), 7.47–7.52 (m, 2H); ^13^C-NMR (DMSO-*d*_6_), δ: 168.98, 159.15, 137.32, 136.14, 134.34, 131.02, 130.97, 129.22, 127.62, 126.06, 124.68, 123.32, 123.13, 120.42, 119.44, 118.46, 107.87; HR-MS: [M − H]^+^ calculated 417.90727 *m*/*z*, found 417.90927 *m*/*z*.

*N-(2,6-Dibromophenyl)-1-hydroxynaphthalene-2-carboxamide* (**19**). Yield 64%; m.p. 212–216 °C; HPLC purity 98.36%; IR (cm^−1^): 3283, 1633, 1616, 1595, 1558, 1522, 1498, 1465, 1441, 1429, 1408, 1382, 1325, 1281, 1209, 1191, 1152, 944, 793, 763, 720, 697; ^1^H-NMR (DMSO-*d*_6_), δ: 13.89 (s, 1H), 10.81 (s, 1H), 8.30 (d, 1H, *J* = 8.6 Hz), 8.11 (d, 1H, *J* = 8.9 Hz), 7.93 (d, 1H, *J* = 8.2 Hz), 7.83 (d, 2H, *J* = 8.2 Hz), 7.69 (ddd, 1H, *J* = 8.1, *J* = 6.9, *J* = 1.2 Hz), 7.60 (ddd, 1H, *J* = 8.1, *J* = 7.0, *J* = 1.0 Hz), 7.50 (d, 1H, *J* = 8.9 Hz), 7.30 (t, 1H, *J* = 8.1 Hz); ^13^C-NMR (DMSO-*d*_6_), δ: 169.70, 160.19, 136.12, 134.85, 132.34, 130.87, 129.31, 127.55, 126.08, 124.59, 124.55, 123.03, 122.72, 118.16, 106.46; HR-MS: [M − H]^+^ calculated 417.9727 *m*/*z*, found 417.90894 *m*/*z*.

*1-Hydroxy-N-(2,4,6-tribromophenyl)naphthalene-2-carboxamide* (**20**). Yield 77%; m.p. 115–118 °C; HPLC purity 99.18%; IR (cm^−1^): 3266, 1610, 1596, 1520, 1500, 1467, 1440, 1387, 1371, 1321, 1273, 1256, 1209, 1153, 1089, 943, 854, 805, 793, 764, 744, 721, 697; ^1^H-NMR (DMSO-*d*_6_), δ: 13.79 (br. s, 1H), 10.81 (s, 1H), 8.30 (d, 1H, *J* = 8.7 Hz), 8.13 (s, 2H), 8.08 (d, 1H, *J* = 8.7 Hz), 7.93 (d, 1H, *J* = 8.2 Hz), 7.67–7.72 (m, 1H), 7.58–7.63 (m, 1H), 7.50 (d, 1H, *J* = 8.7 Hz); ^13^C-NMR (DMSO-*d*_6_), δ: 169.62, 160.25, 136.17, 134.71, 134.48, 129.42, 127.60, 126.16, 125.44, 124.55, 123.07, 122.69, 121.88, 118.26, 106.37; HR-MS: [M − H]^+^ calculated 495.81777 *m*/*z*, found 495.82037 *m*/*z*.

*N-(2-Bromo-4-chlorophenyl)-1-hydroxynaphthalene-2-carboxamide* (**21**). Yield 69%; m.p. 182–184 °C; HPLC purity 99.02%; IR (cm^−1^): 3404, 1631, 1575, 1520, 1503, 1471, 1453, 1414, 1387, 1328, 1296, 1271, 1234, 1208, 1140, 1087, 1033, 1023, 941, 858, 832, 822, 796, 788, 758, 733, 706, 694; ^1^H-NMR (DMSO-*d*_6_), δ: 13.72 (s, 1H), 10.71 (s, 1H), 8.31 (d, *J* = 8.3 Hz, 1H), 8.07 (d, *J* = 8.8 Hz, 1H), 7.91–7.94 (m, 2H), 7.70 (d, *J* = 8.6 Hz, 1H), 7.66–7.70 (m, 1H), 7.55–7.62 (m, 2H), 7.49 (d, *J* = 8.8 Hz, 1H); ^13^C-NMR (DMSO-*d*_6_), δ: 169.30, 159.48, 136.10, 134.88, 132.04, 131.77, 130.12, 129.21, 128.37, 127.58, 126.04, 124.65, 123.13, 123.10, 121.59, 118.32, 107.49; HR-MS: [M + H]^+^ calculated 375.97344 *m*/*z*, found 375.97415 *m*/*z*.

*N-(4-Bromo-2-chlorophenyl)-1-hydroxynaphthalene-2-carboxamide* (**22**). Yield 56%; m.p. 186–187 °C; HPLC purity 98.84%; IR (cm^−1^): 3425, 1628, 1588, 1575, 1520, 1502, 1471, 1456, 1409, 1390, 1382, 1359, 1327, 1299, 1236, 1210, 1168, 1149, 1078, 1048, 1022, 943, 868, 811, 796, 786, 760, 735, 711, 698; ^1^H-NMR (DMSO-*d*_6_), δ: 13.63 (s, 1H), 10.73 (s, 1H), 8.32 (d, *J* = 8.3 Hz, 1H), 8.07 (d, *J* = 9.1 Hz, 1H), 7.93 (d, *J* = 8.3 Hz, 1H), 7.90 (d, *J* = 2.0 Hz, 1H), 7.63–7.71 (m, 3H), 7.57–7.62 (m, 1H), 7.49 (d, *J* = 8.8 Hz, 1H); ^13^C-NMR (DMSO-*d*_6_), δ: 169.06, 159.23, 136.12, 133.81, 131.85, 130.79, 130.77, 129.89, 129.19, 127.60, 126.04, 124.67, 123.27, 123.11, 119.30, 118.42, 107.76; HR-MS: [M + H]^+^ calculated 375.97344 *m*/*z*, found 375.97415 *m*/*z*.

*N-(5-Bromo-2-chlorophenyl)-1-hydroxynaphthalene-2-carboxamide* (**23**). Yield 35%; m.p. 178–180 °C; HPLC purity 99.26%; IR (cm^−1^): 3422, 1632, 1582, 1524, 1503, 1470, 1404, 1388, 1360, 1323, 1288, 1271, 1260, 1240, 1207, 1165, 1151, 1087, 1080, 1041, 1033, 942, 866, 796, 784, 755, 727, 715, 705; ^1^H-NMR (DMSO-*d*_6_), δ: 13.48 (br. s, 1H), 10.82 (s, 1H), 8.33 (d, *J* = 8.3 Hz, 1H), 8.07 (d, *J* = 7.3 Hz, 1H), 8.05 (s, 1H), 7.93 (d, *J* = 8.1 Hz, 1H), 7.66–7.71 (m, 1H), 7.53–7.63 (m, 3H), 7.51 (d, *J* = 8.8 Hz, 1H); ^13^C-NMR (DMSO-*d*_6_), δ: 168.76, 158.86, 136.16, 135.82, 131.23, 130.38, 130.33, 129.19, 128.40, 127.63, 126.07, 124.71, 123.47, 123.15, 119.72, 118.59, 108.18; HR-MS: [M + H]^+^ calculated 375.97344 *m*/*z*, found 375.97421 *m*/*z*.

*N-(4-Bromo-3-chlorophenyl)-1-hydroxynaphthalene-2-carboxamide* (**24**). Yield 58%; m.p. 203–205 °C; HPLC purity 99.53%; IR (cm^−1^): 3429, 1627, 1599, 1572, 1520, 1504, 1464, 1406, 1381, 1321, 1293, 1272, 1245, 1207, 1150, 1139, 1114, 1086, 1012, 935, 869, 794, 783, 757, 727, 695; ^1^H-NMR (DMSO-*d*_6_), δ: 13.61 (s, 1H), 10.67 (s, 1H), 8.31 (d, *J* = 8.3 Hz, 1H), 8.12 (d, *J* = 2.3 Hz, 1H), 8.07 (d, *J* = 9.1 Hz, 1H), 7.91 (d, *J* = 8.1 Hz, 1H), 7.80 (d, *J* = 8.8 Hz, 1H), 7.66–7.72 (m, 2H), 7.56–7.61 (m, 1H), 7.48 (d, *J* = 8.8 Hz, 1H); ^13^C-NMR (DMSO-*d*_6_), δ: 169.58, 159.92, 138.51, 136.08, 133.76, 132.92, 129.32, 127.50, 126.04, 124.58, 123.10, 122.96, 122.89, 121.83, 118.01, 116.08, 107.45; HR-MS: [M + H]^+^ calculated 375.97344 *m*/*z*, found 375.97397 *m*/*z*.

### 3.3. Study of Inhibition of Photosynthetic Electron Transport (PET) in Spinach Chloroplasts

Chloroplasts were prepared from spinach (*Spinacia oleracea* L.) according to Masarovicova and Kralova [49]. The inhibition of photosynthetic electron transport (PET) in spinach chloroplasts was determined spectrophotometrically (Genesys 6, Thermo Scientific, USA), using an artificial electron acceptor 2,6-dichlorophenol-indophenol (DCIPP) according to Kralova et al. [50], and the rate of photosynthetic electron transport was monitored as photoreduction of DCPIP. The measurements were carried out in phosphate buffer (0.02 M, pH 7.2) containing sucrose (0.4 M), MgCl_2_ (0.005 M) and NaCl (0.015 M). The chlorophyll content was 30 mg/L in these experiments, and the samples were irradiated (~100 W/m^2^ with 10 cm distance) with a halogen lamp (250 W) using a 4-cm water filter to prevent warming of the samples (suspension temperature 22 °C). The studied compounds were dissolved in DMSO due to their limited water solubility. The applied DMSO concentration (up to 4%) practically did not affect the photochemical activity in spinach chloroplasts. The inhibitory efficiency of the studied compounds was expressed by IC_50_ values, i.e., by molar concentration of the compounds causing a 50% decrease in the oxygen evolution rate relative to the untreated control. The comparable IC_50_ value for a selective herbicide 3-(3,4-dichlorophenyl)-1,1-dimethylurea, DCMU (Diuron^®^, Merck, Darmstadt, Germany) was about 1.9 μM. The results are summarized in Table 1.

### 3.4. Study of Fluorescence of Chlorophyll a and Aromatic Amino Acids in Spinach Chloroplasts

The fluorescence emission spectra of chlorophyll *a* (Chl*a*) and aromatic amino acids in spinach chloroplasts were recorded on fluorescence spectrophotometer F-2000 (Hitachi, Tokyo, Japan) using excitation wavelength λ_ex_ = 436 nm for monitoring the fluorescence of Chl*a* and λ_ex_ = 275 nm for monitoring the fluorescence of aromatic amino acids, excitation slit 20 nm and emission slit 10 nm. The samples were kept in the dark for 2 min prior to the measurement. The phosphate buffer used for dilution of the chloroplast suspension was the same as described above. Due to low aqueous solubility, the compounds were added to a chloroplast suspension in DMSO solution. The DMSO concentration in all samples was the same as in the control (10%). The chlorophyll concentration in chloroplast suspension was 10 mg/L.

## 4. Conclusions

Series of seventeen new di- and trichlorinated and brominated 1-hydroxynaphthalene-2-carboxanilides was prepared and characterized. These compounds were completed by seven recently published unsubstituted and monochlorinated and brominated derivatives, and all the compounds were tested for their in vitro activity related to the inhibition of photosynthetic electron transport (PET) in spinach (*Spinacia oleracea* L.) chloroplasts. *N*-(3,5-dichlorophenyl)-(**13**), *N*-(4-bromo-3-chlorophenyl)-(**24**), *N*-(2,5-dibromophenyl)-(**18**) and 1-hydroxy-*N*-(3,4,5-trichloro-phenyl)naphthalene-2-carboxamide (**16**) were the most potent PET inhibitors with IC_50_ = 5.2, 6.7, 7.6 and 8.0 µM, respectively. In general, it can be stated that the chlorinated derivatives demonstrated higher potency than the bromine derivatives. The inhibitory activity of these compounds depends on the position and the type of halogen substituents, e.g., all 2,4-disubstituted compounds **9**, **17**, **21**, **22** and 2,3-disubstituted compound **8** showed limited aqueous solubility resulting in moderate PET-inhibiting activity compared with that of 2,5-, 2,6-, 3,4- or 3,5-disubstituted derivatives or other investigated compounds. It can be stated that the PET inhibition increases with increasing lipophilicity for mono- and disubstituted analogues, while an opposite trend is observed for trisubstituted derivatives. Thus, it seems that for PET-inhibiting activity, a lipophilicity optimum is in the range of log *P* from 6.01 to 6.28. On the other hand, the PET inhibition decreases with increasing electron-withdrawing properties of individual anilides, and this trend is similar for all the series. Interactions of the studied compounds with chlorophyll *a* and aromatic amino acids present in pigment–protein complexes mainly in PS II were documented by fluorescence spectroscopy. The section between P_680_ and plastoquinone Q_B_ in the PET chain occurring on the acceptor side of PS II can be suggested as the site of action of the compounds.

## Figures and Tables

**Table 1:**
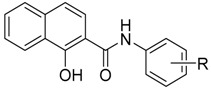
Structure of ring-substituted 1-hydroxynaphthalene-2-carboxanilides **1**–**24**, calculated values of log *P*, electronic σ parameters of anilide (Ar) substituents and IC_50_ [µM] values related to photosynthetic electron transport (PET) inhibition in spinach chloroplasts in comparison with 3-(3,4-dichlorophenyl)-1,1-dimethylurea (DCMU) standard.

Comp.	R	log *P ^a^*	σ_(Ar)_ *^a^*	PET Inhibition IC_50_ [µM]
**1** *^b^*	H	4.52	0.60	31.3
**2** *^b^*	2-Cl	5.02	1.05	29.4
**3** *^b^*	3-Cl	5.25	0.85	7.9
**4** *^b^*	4-Cl	5.24	0.75	10.8
**5** *^b^*	2-Br	5.06	0.97	61.9
**6** *^b^*	3-Br	5.39	0.86	8.2
**7** *^b^*	4-Br	5.28	0.74	9.6
**8**	2,3-Cl	5.76	1.22	235.3
**9**	2,4-Cl	5.78	1.12	531.0
**10**	2,5-Cl	5.82	1.22	59.6
**11**	2,6-Cl	5.52	1.33	100.5
**12**	3,4-Cl	5.99	1.19	13.3
**13**	3,5-Cl	6.01	1.11	5.2
**14**	2,4,5-Cl	6.31	1.56	153.2
**15**	2,4,6-Cl	6.15	1.48	10.6
**16**	3,4,5-Cl	6.28	1.46	8.0
**17**	2,4-Br	5.90	1.11	505.1
**18**	2,5-Br	5.81	1.23	7.6
**19**	2,6-Br	5.67	1.33	78.0
**20**	2,4,6-Br	6.34	1.47	45.0
**21**	2-Br-4-Cl	5.80	1.12	277.5
**22**	4-Br-2-Cl	5.88	1.11	171.0
**23**	5-Br-2-Cl	5.82	1.23	60.3
**24**	4-Br-3-Cl	6.02	1.19	6.7
**DCMU**	-	-	-	1.9

*^a^* calculated using ACD/Percepta ver. 2012 (Advanced Chemistry Development, Toronto, ON, Canada); *^b^* published in Gonec et al. [11].
